# Association Between Vitamin D and Cortisol Concentrations Among Pregnant Women

**DOI:** 10.3390/nu17193055

**Published:** 2025-09-25

**Authors:** Kenneth S. Addae, Isaac Agbemafle, Guangyu Zhu, Alyssa Abreu, Zachary Jacques, Bridget Owens, Christopher Vatral, Brie M. Oaks

**Affiliations:** 1 Department of Nutrition, College of Health Sciences, University of Rhode Island, Fogarty Hall 41 Lower College Road, Room 125, Kingston, RI 02881, USA; isaac.agbemafle@uri.edu (I.A.); amabreu@uri.edu (A.A.); zrjacques@uri.edu (Z.J.); christopher.vatral@gmail.com (C.V.); boaks@uri.edu (B.M.O.); 2 Department of Computer Science and Statistics, College of Arts and Sciences, University of Rhode Island, Tyler Hall, 9 Greenhouse Road, Rm 264, Kingston, RI 02881, USA; guangyuzhu@uri.edu; 3 Military Performance Division, U.S. Army Research Institute of Environmental Medicine, 10 General Greene Ave, Natick, MA 01760, USA; bridget.a.owens.mil@health.mil

**Keywords:** vitamin D, cortisol, pregnancy

## Abstract

**Background:** Vitamin D deficiency (VDD) and high circulating cortisol during pregnancy have each been linked to adverse maternal and child outcomes, such as pre-term birth. However, limited research has explored the association between vitamin D concentration and cortisol concentration during pregnancy. Our objective for this study was to determine the association between vitamin D and cortisol concentrations among pregnant women. **Methods**: The Prenatal Health and Nutrition (PHAN) study was a pilot cross-sectional study conducted in targeted areas in Rhode Island which included 30 healthy pregnant women. Data collection included sociodemographic characteristics of participants, substance use, perceived stress, food insecurity, and dietary assessments. Additionally, blood samples were collected to estimate vitamin D status (25(OH)D). Participants provided 10 saliva samples over a two-day period, which were used to estimate cortisol concentrations using a competitive Enzyme-Linked Immunosorbent Assay (ELISA). Linear and logistic regression models were used to analyze the association between vitamin D status and cortisol concentrations. **Results**: Mean ± SD age was 29 ± 6 years, and pre-pregnancy BMI was 28 ± 6 kg/m^2^. The mean ± SD of serum 25(OH)D concentration was 24 ± 9 ng/mL. The prevalence rates of VDD (<20 ng/mL) and vitamin D insufficiency (20–29 ng/mL) were 40% and 43%, respectively. In the unadjusted and adjusted linear regression models, there was no significant association between vitamin D status and cortisol concentration parameters such as AUCg (adjusted model β: 2.987, 95% CI: −7.269, 13.244, *p* = 0.57). Additionally, no significant association was observed in the logistic regression model. **Conclusions**: There were no significant associations between vitamin D and cortisol concentrations. Our study revealed a high prevalence of vitamin D deficiency and insufficiency. We advocate for screening of vitamin D status among pregnant women and the intake of vitamin D supplements. Future studies should explore prospective cohorts to examine the association between vitamin D and cortisol concentrations among pregnant women.

## 1. Introduction

Cortisol is the product of the hypothalamic–pituitary–adrenal (HPA) axis. Cortisol secretion follows a strong circadian rhythm, peaking in the early morning, gradually decreasing throughout the day, and reaching its lowest levels at night. Although cortisol levels naturally rise significantly during pregnancy, physical or mental stress can further activate the hypothalamus–pituitary–adrenal (HPA) axis, leading to an increase in circulating cortisol [1]. The primary role of cortisol is to help the body respond and manage stress and regulate other important metabolic processes in the body, such as glucose metabolism. High circulating cortisol concentrations in pregnant women have been shown to be associated with pre-term birth [2], which is the leading cause of child mortality under 5 years [3], impaired growth [4], and cognitive development of the fetus [5]. Therefore, managing cortisol during pregnancy is crucial to ensure optimal maternal and fetal health outcomes. As a physiological process to regulate circulating cortisol in the fetus, the placenta produces an enzyme called 11-β-hydroxysteroid dehydrogenase 2 (11-β-HSD-2), which converts cortisol to an inactive metabolite called cortisone [6]. However, several factors have been shown to decrease the production of this enzyme, including poor nutritional status [7]. Vitamin D deficiency has particularly been proposed to decrease the production of this enzyme [8]. However, the mechanism and associations are not clearly understood.

In a normal pregnancy, cortisol levels increase by up to 300% compared to non-pregnant levels by the third trimester [9]. This is attributed to the corticotropin-releasing hormone (CRH) in the hypothalamus that is released during stress, which is under a negative feedback loop, and the placenta CRH (pCRH), which is under a positive feedback loop [10,11]. With a sharp rise in cortisol levels during pregnancy, it is essential to investigate factors like vitamin D, which some studies in non-pregnant populations suggest may influence cortisol levels [8,12,13]. VDD, defined as a 25-hydroxyvitamin D concentration (25OHD) below 20 ng/mL, is recognized as a global health problem, affecting over 1 billion people worldwide [14,15]. VDD is considered an important public health priority given its adverse health effect. Studies have reported a high prevalence of VDD among pregnant women, ranging from 26 to 95% globally [16]. A systematic review paper on the global summary of maternal and newborn vitamin D status revealed a 64% prevalence in VDD among pregnant women in the US [17]. Most humans obtain vitamin D through exposure of the skin to sunlight [18]. Few foods naturally contain vitamin D, and therefore, popular food products such as milk are fortified with vitamin D [19]. A dietary guideline paper suggests that pregnant women require at least 600 IU/d of vitamin D and recognizes that at least 1500–2000 IU/d of vitamin D may be needed to maintain a blood level of 25(OH)D above 30 ng/mL [20].

Geographically, New England states are situated in the northern latitudes, generally spanning from 43° N to 47° N. This is of concern since a study highlights the low synthesis of vitamin D in latitudes above 33° N [18]. A study of Hispanic and African American adults in Boston reported a 50% prevalence of vitamin D deficiency in the New England states, while another study reported that 48% of white preadolescent girls in Maine were deficient in vitamin D [21,22]. To the best of our knowledge, limited studies have explored the association between vitamin D and cortisol concentrations in pregnant women in New England, specifically Rhode Island. A study conducted in Sweden among pregnant women revealed that serum cortisol and vitamin D statuses were independently associated with blood pressure in pregnancy [23], and as part of their secondary analysis, they explored the association between serum cortisol and vitamin D statuses among pregnant women and found no significant difference. This study, however, explored serum cortisol rather than salivary cortisol, which is a better indicator of unbound cortisol [24].

Additionally, in a non-pregnant population, no significant association was found between vitamin D status and cortisol among ice hockey players [25] and among men practicing strength training that were provided with vitamin D supplementation and a combination of different carbohydrates and fats [13]. Conversely, a randomized placebo-controlled preliminary study that examined the effect of vitamin D supplementation and cardiovascular risk factors found a decrease in 11β-HSD1 activity, which has been shown to decrease the cortisol/cortisone ratio [12]. Similarly, a vitamin D supplementation study among individuals with Cushing’s disease revealed that serum vitamin D was lower in patients with Cushing’s disease than in the controls who did not receive vitamin D supplements [26]. An animal experimentation model revealed that prenatal vitamin D deficiency leads to an increase in maternal corticosterone [27]. VDD among pregnant women has also been linked to an increased risk of maternal and child health disparities [16]. A clinical research study showed that pregnant women who take prenatal vitamin supplements remain at risk for vitamin D deficiency [28]. With evidence-based research on the detrimental effects of VDD and high circulating cortisol among pregnant women, it is very crucial to explore the association between vitamin D and cortisol concentrations to better understand whether vitamin D deficiency could act as a stressor influencing cortisol production. We hypothesize that vitamin D deficiency, a form of micronutrient malnutrition, is associated with increased salivary cortisol among pregnant women in the Prenatal Nutrition and Health (PHAN) study.

## 2. Materials and Methods

### 2.1. Participants and Setting

A total of 30 participants were enrolled in a pilot study, the Prenatal Health and Nutrition (PHAN) study, which aimed to explore how nutrition, stress, and blood lead (Pb) concentrations are related among pregnant women in Rhode Island. However, in reference to this manuscript, the variables of interest were vitamin D status and stress (cortisol). Participants were recruited through (1) flyers posted in the targeted areas of Providence, Woonsocket, Central Falls, and Pawtucket; (2) text blasts sent out by the Women Infants and Children (WIC) Nutrition Program to women enrolled in WIC in Rhode Island; and (3) in-person recruitment at WIC prenatal offices in the targeted areas of the study. Recruitment materials included a direct link to an online prescreening questionnaire to determine eligibility. To participate in the PHAN study, pregnant women had to be 18 years or older, pregnant with a single child, at risk of lead (Pb) exposure, and able to read and write English. Pb exposure screening assessed several potential sources, including residence location; recent home renovations; length of residence; use of imported remedies, foods, spices, ceramics, or cosmetics; and occupational exposures of participants or household members. The questionnaire also inquired about prior Pb diagnoses within the household and whether household water had been tested and confirmed to be Pb-free. Participants became eligible for the study if they related to at least two items in Pb screening. However, those ultimately enrolled had Pb levels below the limit of detection. Pregnant women were excluded from the study if they were diagnosed with endocrine disorders such as Cushing’s syndrome, had underlining health conditions such as diabetes, or were on steroid-based medications. If recruited in person, written informed consent was obtained at the time of recruitment. If recruited by study flyer or text blast, written informed consent was obtained by electronic signature after the consent form was reviewed with a research assistant virtually before the start of the surveys.

### 2.2. Study Design

This study employed a cross-sectional design. Eligible participants were scheduled for a 1 h 30 min zoom meeting. This meeting included surveys on socio-demographic characteristics, food security, and perceived stress. Participants were also required to complete an Automated Self-Administered 24 h (ASA24) [29] recall online to capture the foods consumed the day before the ASA24 recall was taken. This captures all foods, drinks, and dietary supplements consumed from midnight to midnight of the day before. At the end of the meeting, participants were instructed to complete the diet ASA24 one additional time at home on their own. Participants were scheduled for a blood draw and a time to receive their saliva kit if they had not already received it at recruitment. The saliva kit was used to collect saliva samples at home over the course of 2 days. These days could either be consecutive or non-consecutive days. After the online interview, participants received a USD 50 Amazon gift card. A second Amazon gift card worth the same price was provided at their appointed time for the blood drawing, at which time they returned their collected saliva samples.

### 2.3. Salivary Collection and Cortisol Analysis

Participants provided 10 samples over a 2-day period. Participants were provided with labeled salivettes and instructed to collect their saliva immediately upon waking and before getting out of bed; 30, 45, and 60 min after waking; and 1 hour before going to bed. Participants received a record sheet to record the dates and times of waking and saliva collection for each measurement. Participants were also given an instruction sheet which instructed participants not to brush their teeth or eat one hour prior to saliva collection and to store their saliva samples in the freezer immediately after collection. If the samples could not be stored in the freezer immediately, participants were instructed to store them in the fridge for up to 24 h until they could be stored in the freezer. At the scheduled blood draw at the Quest Diagnostic laboratory, the saliva kits were collected from the study subjects and transported to the Oaks laboratory at the University of Rhode Island (URI). Samples were stored in a freezer at a temperature of −80 °C. Saliva samples were centrifuged at a speed of 1500 revolutions for 15 min before analysis and analyzed in duplicate using a high-sensitivity salivary cortisol enzyme immunoassay using salivary assay kits ordered from salimetrics. All samples with intra-assay coefficients of variation greater than 10% were repeated. The intra-assay coefficients of variation were below 4%, and inter-assay coefficients were below 15%. The average cortisol over the 2-day period for each time point was used in analysis, and if participants were missing a sample for one day, a sample from the second day was used to preserve data.

### 2.4. Perceived Stress Questionnaire

Perceived stress was measured during an online Zoom meeting using the 10-item Perceived Stress Scale (PSS) [30] to assess the degree to which an individual perceives situations in life as being stressful. The items are rated on a 5-point Likert scale, which ranges from 0 (never) to 4 (very often). Scores were obtained by reversing the scores on the four positive items and then summing the scores across all 10 items. Scores ranged from 0 to 40. Higher scores are associated with greater perceived stress.

### 2.5. Vitamin D Analysis (25(OH)D)

All blood tests and measurements were performed by Quest Diagnostics. Total 25-hydroxyvitamin D is the major circulating form of vitamin D and the gold standard for measuring serum vitamin D status [31]. Total 25-hydroxyvitamin D was measured using the DiaSorin LIAISON^®^ chemiluminescence immunoassay (DiaSorin Inc., Saluggia, Italy). The assay’s detection range is 4.0–150 ng/mL. The laboratory used the National Institute of Standards and Technology (NIST) level 1 protocol for quality control for each run. The inter-assay coefficient of variations were 7.6% at a 25OHD level of 47.8 nmol/L and 6.9% at a 25OHD level of 83.0 nmol/L. The assay is credited by the Vitamin D External Quality Assessment Scheme (DEQUAS).

### 2.6. Sociodemographic and Substance Use Data

Sociodemographic data was collected during this study. This included participants’ age, level of education, current occupation, household income, and race. Substance use data included smoking status before and during pregnancy through either cigarettes, vaping nicotine, or vaping marijuana, and alcohol intake before and during pregnancy.

### 2.7. U.S Household Food Security Survey Module (HFSSM)

The U.S HFSSM has been validated through studies that have shown that the 18-item and 6-item survey modules provide reliable and valid measures of household food security [32,33]. The updated food security questionnaire with screeners was used to determine the food security status of participants [34]. This questionnaire is partitioned in 3 stages for participants without children and 5 stages for participants with a child or children.

### 2.8. Automated Self-Administered 24-h (ASA-24) Recall

A self-administered 24-h recall was used to capture foods, beverages, and dietary supplements consumed by participants a day prior to the assessment. This recall was conducted twice, and averages were used for the two-day period. The Automated Self-Administered 24-h (ASA24) recall is a free web-based dietary assessment tool. It was developed by the National Cancer Institute (NCI) of the National Institutes of Health (NIH). It incorporates food codes from the U.S. Department of Agriculture (USDA) and Food and Nutrient Database for Dietary Studies (FNDDS).

### 2.9. Data Analysis

Statistical analysis was performed using R programing R-4.3.2 for Windows. Sociodemographic data was analyzed into frequencies and percentages for categorical variables and means and standard deviations were used for continuous variables. The U.S. Household Food Security Survey Module was analyzed by assigning scores based on affirmative responses to questions within the module. The HFSSM scoring rubric was used to categorize households into food security categories (food secure and food insecure). The ASA-24 automatically analyzes the nutrient intake of the participants.

The data analysis assessed potential covariates that could influence the outcome of interest, cortisol. These include demographics of age [35], race and ethnicity [36], gestational age [37,38], and pre-pregnancy BMI [37]. These covariates, according to previous research, have been shown to influence cortisol. Of note, we examined other potential covariates, such as dietary habits using the ASA24, perceived stress, food security, and socioeconomic status, which were available for all 30 participants. We did not include these as covariates in the final models as they did not improve model fitness due to the sample size used in the study [39]. The selected covariates were once most citated in the literature. Pearson’s correlation was performed as a secondary analysis to assess the correlations between PSS, HFSSM, cortisol, and vitamin D.

Statistical analysis commenced with data cleaning in Microsoft Excel 2024. Both linear and logistic regression were performed. In linear regression, the variables of interest remained continuous, while in logistic regression, variables were treated as categorical. Outliers were not removed because of their biological plausibility. The Shapiro–Wilk test was used to test for normality, linearity, and homoscedasticity. Raw cortisol values were log-transformed to adhere to parametric methods for analysis. In analyzing cortisol, various methods exist to capture cortisol secretion and variability throughout the day, including the diurnal cortisol slope and total cortisol secretion or area under the curve (AUC). For this study, the AUC with respect to ground (AUC_g_), cortisol awakening response (CAR), and all the time point measures were used to assess cortisol concentration [40]. AUC_g_ represents the total cortisol output over the specific time periods, that is, waking cortisol, cortisol + 30 min, cortisol + 45 min, cortisol + 60 min, and cortisol before retiring to bed. The AUC_g_ is then calculated using the trapezium method to sum up cortisol across multiple time points. The formula is applied to the average of the two consecutive cortisol values and multiplied by the time interval between them [41]. Additionally, cortisol awakening response (CAR) quantifies the increase in cortisol levels within the first 30–45 min after awakening. It is calculated by finding the difference in cortisol concentration after 30 or 45 min and waking cortisol [42]. This study used 30 min after waking to calculate the CAR because that was when the highest peak in cortisol occurred in our sample Adjusted and unadjusted linear regression models were used to determine the association between vitamin D (25OHD) and all the measures of cortisol, such as the AUC_g_. Adjusted and unadjusted logistic regression were models used to determine the association between vitamin D and cortisol measures. Vitamin D status was categorized as deficient (<20 ng/mL), insufficient (20–29 ng/mL), or optimal (>/=30 ng/mL) based on the cutoffs from the Endocrine Society, which are targeted towards vulnerable groups such as pregnant women [43]. Also, a prospective study among pregnant women revealed that serum concentrations >30 ng/mL significantly lowered the risk of adverse birth outcomes [44]. To conduct an analysis based on binary logistic regression, individuals categorized as deficient and insufficient were grouped together as sub-optimal in comparison with the optimal group. As no clinical cutoff exists for identifying elevated cortisol during pregnancy, the median split of cortisol was used to categorize cortisol values as high and low among participants of this study [5]. Some of the figures for the results were analyzed using Graph pad 10.2.2.

## 3. Results

### 3.1. Baseline Characteristics

As shown in Table 1, the mean age of the study participants was 28 ± 6 years. Participants had an average pregnancy BMI of 27.9 ± 6.2 kg/m^2^. One third of the participants had normal BMI, which is considered a BMI categorization within the range of 18.5 and 24.9 kg/m^2^. Conversely, 6.7% of the participants were classified as underweight. In reference to socioeconomic status, 90% of the study sample reported an annual household income below USD 60,000. In the racial and ethnic distribution category, most participants self-identified as being white, followed by Black/African American. A total of 46.7% of the participants identified as Hispanic, Latinx, or Spanish regarding ethnicity. Most of the participants were primiparous mothers. The mean vitamin D and cortisol AUC_g_ concentrations were 23.7 ± 9 ng/mL and 659.5 ± 212.1 nmol/L, respectively. Table 2, generally, all pregnant women reported not vaping or smoking any sort of substance during pregnancy. Most participants (93.3%) reported not consuming alcohol during pregnancy. Overall, substance and alcohol use before pregnancy was reported to be relatively low 3 months before pregnancy.

**Figure 1:**
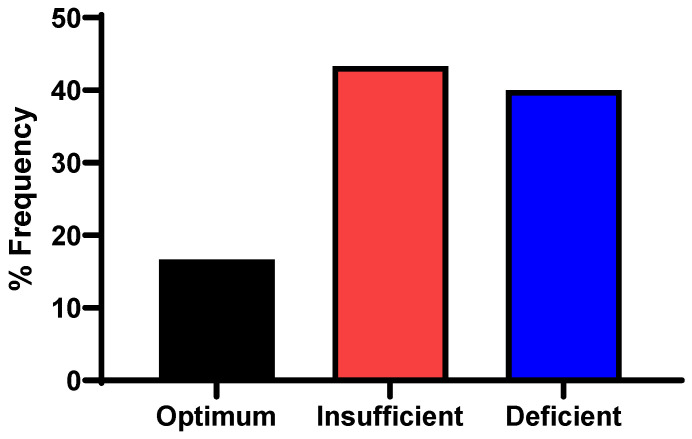
Prevalence of vitamin D status among study participants.

Figure 1 shows the prevalence rates of VDD (<20 ng/mL) and vitamin D insufficiency (20–29 ng/mL) as 40% and 43%, respectively.

**Figure 2:**
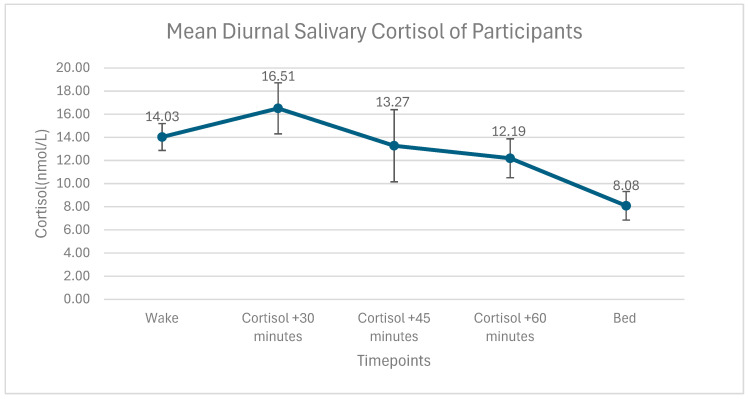
Mean diurnal salivary cortisol of participants.

Figure 2 shows the circadian rhythm of cortisol, with cortisol levels typically spiking 30 min after waking and gradually declining. The figure also shows the error bars around the standard deviation of the average cortisol timepoints.

### 3.2. Association Between Vitamin Concentration and Cortisol Concentration Among Pregnant Women

There were no significant associations between vitamin D and all measures of cortisol in Table 3.

**Figure 3:**
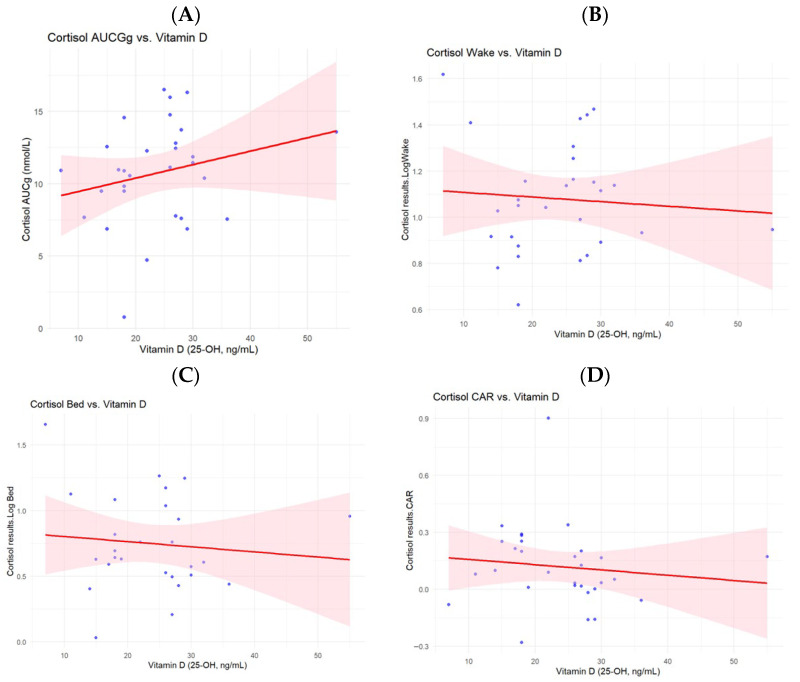
Visualization of the relationship between 25(OH)D and cortisol metrices of AUC, waking and bedtime cortisol levels, and CAR obtained using linear regression model.

Figure 3 shows the trends in the relationship between vitamin D and cortisol concentrations with the different cortisol measures. These cortisol indicators were visualized to observe the trend in the data set as exploratory since this was a pilot study. The scatter plot displays individual data points (blue dots), with a fitted regression line (red) and shaded confidence. Figure 3A shows a weak positive trend using (AUC_g_). Figure 3B–D show weak negative trends between vitamin D and Cortisol Wake, Cortisol Bed and CAR respectively. 

### 3.3. Odds Ratios Associated with Vitamin D Concentration and Cortisol Concentratoion Among Pregnant Women

Overall Table 4 had no significant association between vitamin D concentration and cortisol concentration. In the unadjusted model, the odds ratio for the association between vitamin D and cortisol AUC_g_ was 1.63, with a 95% confidence interval of 0.23 to 2.04 and a *p*-value of 0.63.

### 3.4. Pearson’s Correlation Matrix

In Table 5, the correlation analysis showed a moderate positive association between perceived stress (PSS) and household food insecurity (HFSSM) (r = 0.44), suggesting that higher stress scores were linked to greater food insecurity. Cortisol (AUCg) showed a weak positive correlation with HFSSM (r = 0.40) and very weak correlations with PSS (r = 0.13) and vitamin D (r = 0.23). HFSSM was weakly correlated with vitamin D (r = 0.28), and PSS had a minimal correlation with vitamin D (r = 0.15).

## 4. Discussion

In this study, we tested the hypothesis that pregnant women who are vitamin D deficient, that is, women with a vitamin D status (25(OH)D) of less than 20 ng/mL, will have a relatively higher concentration of cortisol in their saliva. Based on our statistical analysis, we accepted the null hypothesis, which shows that our study did not find a significant association between vitamin D and cortisol. By visualizing the trends in the linear regression models of AUC_g_, CAR, waking cortisol, and bedtime cortisol, which have each been used as a reliable marker for cortisol dysregulation [46], we only observed a weak positive correlation between vitamin D and AUC_g_. However, a negative weak correlation was seen among vitamin D and CAR, vitamin D and waking cortisol, and vitamin D and bedtime cortisol. Although these trends are statistically insignificant, this pilot study serves as a baseline for further investigations as our data shows large confidence intervals in most of the models, potentially suggesting that sample size issues increase variability. Generally, as seen in the table, β-coefficients werevery small, indicating that the effect is likely to be weak. In the correlation matrix in Table 5, a similar weak correlation is seen between vitamin D and cortisol concentrations.

**Figure A1:**
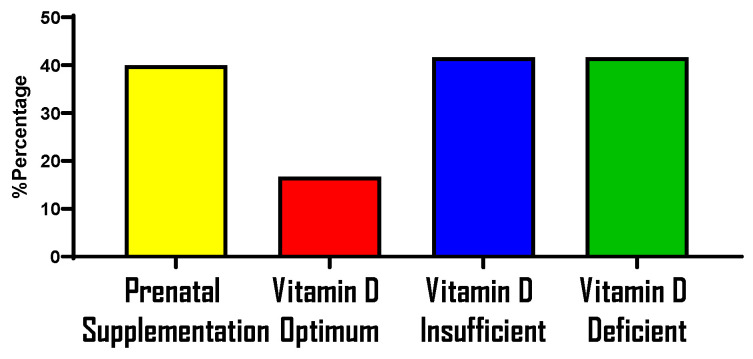
Bar graph showing the reported percentages of pregnant women that were taking prenatal supplementation and a stratified representation of their vitamin D status.

**Figure A2:**
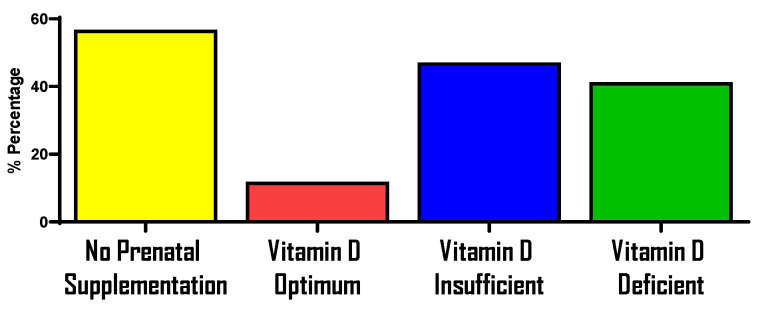
Bar graph showing the reported percentages of pregnant women that were not taking prenatal supplementation and a stratified representation of their vitamin D status.

In this pilot study, 83.3% of the women were found to have suboptimal vitamin D status. These findings are consistent with the existing literature that quantifies VDD as a global public health problem that is very common in pregnancy [47,48,49,50,51,52,53]. Our study participants were recruited from targeted areas in Rhode Island, U.S. The high prevalence of VDD among pregnant participants aligns with previous research indicating the widespread vitamin D insufficiency among Black and white pregnant women in the Northern United States [54]. Our findings align with the established geographical location being a factor of VDD, thus showing that locations above latitude 35° may have an influence on VDD [55]. In terms of ethnicity, most of our study sample identified as being of Hispanic, Latinx, or Spanish origin. We could draw inferences to the high prevalence of VDD to ethnicity as a study that analyzed data from the National Health and Nutrition Examination Survey within a period showed the second highest prevalence of VDD to be among Hispanics with a 69.2% prevalence rate [56]. In the supplementary figures in Appendix A (Figure A1 and Figure A2) also highlights that most participants remain vitamin D insufficient or deficient, regardless of whether they reported taking prenatal supplements.

In general, the mean cortisol concentrations determined in the study population are like those found in pregnant women. The mean cortisol AUC_g_ for our study was 650.47 ± 212.10 nmol/L, which is like that in a study among pregnant women whose AUC_g_ was 410 ± 120 nmol/min [57]. The cortisol in our study follows the expected diurnal pattern of cortisol. Additionally, cortisol concentrations in our study also follow the expected pattern of free circulating cortisol, increasing in mid-gestation. However, a decline is observed in the third trimester which is contrary to what is observed in literature [9,38]. This may be attributed to the small sample size that is not evenly distributed across pregnancy stages, with most study participants being in the second trimester. To the best of our knowledge, no known study has examined the association of vitamin D concentration and salivary cortisol concentration using vitamin D as a predictor among pregnant women in the U.S. Although a study conducted in Sweden in their secondary analysis explored the association between serum cortisol and vitamin D status among pregnant women and found no significant difference [23]. This study, however, explored salivary cortisol rather than serum cortisol, which is a better indicator of unbound cortisol [37].

In an animal study conducted among pregnant sheep, it was revealed that chronic stress may disrupt covariant fluctuations in vitamin D and cortisol plasma levels during the last trimester of the animals [58]. This could be a plausible reasoning for the integrated mechanisms that affect vitamin D and cortisol, and therefore, their association could be marred. A non-significant association is also observed in a study that examined the association between vitamin D status and cortisol in ice hockey players [25]. In addition, a study conducted among strength trainers that explored the combination of a diversified intake of carbohydrates and fats and supplementation of vitamin D in a diet revealed that these independent predictors did not affect hormones which included cortisol [13]. Although these are different study populations, the results provide us with some form of evidence among these variables. In summary, this pilot revealed a non-significant association between vitamin D concentration and salivary concentration. Thus, vitamin D supplementation during pregnancy may not necessarily be associated with the reduction of cortisol concentration. This could be because of the multiple factors that regulate cortisol and vitamin D.

There are many strengths of this study that should be noted. First, saliva was used to determine cortisol concentration, which is currently deemed the most accurate measurement of bioavailable cortisol. Serum cortisol is mostly bound to binding proteins and is not biologically active, and hair cortisol is a new item for measuring cortisol, representing a method that is still being established. Also, salivary cortisol is non-invasive compared to other biomarkers. Salivary cortisol was analyzed using a competitive ELISA with very good intra and inter assay coefficients. Serum vitamin D was used as the biomarker for measuring vitamin D status. Data collection was performed using standard procedures and questionnaires. Data analysis was performed using robust statistical methods that controlled covariates. Additionally, scheduling blood draws at the nearest Quest Diagnostics center enhanced feasibility by reducing travel burden. This arrangement allowed participants to conveniently complete their blood collection while picking up saliva samples in a single visit, which likely improved compliance and overall study participation.

However, there are limitations in our study that should be noted. The main limitation is the sample size, as this was a pilot study, which potentially underpowered our study. Also, participants enrolled in the study were in different pregnancy stages, which potentially skewed the data even though gestational age was added to our covariate to adjust for pregnancy stages. Furthermore, we acknowledge that this pilot study has a limited generalizability to the entire pregnant population.

## 5. Conclusions

Our pilot cross-sectional study amongst pregnant women in Rhode Island examined the association between vitamin D status and cortisol concentration. However, no significant association was found. Our study revealed a high prevalence of vitamin D deficiency and insufficiency. With evidence-based findings on the impact of VDD on maternal and neonatal outcomes, we advocate for screening of vitamin D status among pregnant women and the intake of vitamin D supplements. Future studies should explore prospective cohorts to examine the association between vitamin D and cortisol concentrations among pregnant women.

## Figures and Tables

**Table 1:** Background characteristics of participants (N = 30).

Participant’s Characteristics	Frequency (%)/Mean ± SD
Age (yr)	28 ± 6
Pre-pregnancy weight (kg)	71.3 ± 16.6
Pre-pregnancy BMI (kg/m^2)^	27.9 ± 6.2
Underweight (BMI < 18.5 kg/m^2^)	2 (6.7)
Normal weight (BMI 18.5–24.9 kg/m^2^)	10 (33.3)
Overweight (BMI 25–29.9 kg/m^2^)	10 (33.3)
Obese (BMI ≥30 kg/m^2^)	8 (26.7)
Annual household income ^a^	
Less than USD 60,000	27 (90)
Over USD 60,000	3 (10)
Highest education attained	
Completed high school and or associate degree	17 (56.7)
Bachelor’s degree or graduate degree	13 (43.3)
Race ^b^	
Asian	1 (3.3)
Black or African American	7 (23.3)
Native Hawaiian or other Pacific Islander	2 (6.7)
White	17 (56.7)
Other race	3 (10)
Ethnicity ^b^	
Hispanic, Latinx, or of Spanish origin	14 (46.7)
Not Hispanic, Latinx, or of Spanish origin	16 (53.3)
Marital status %	
Single	16 (53.3)
Married or in a domestic partnership	13 (43.3)
Divorce/separated	1 (3.3)
Number of children given birth to	
None	16 (53.7)
1–2	10 (33.3)
3–4	4 (13.3)
Cortisol AUC_g_ (nmol/L)	650.5 ± 212.1
Cortisol average at wake time * (AM)	8:20
Cortisol average at bedtime * (PM)	10:22
Weekday cortisol *	19 (74)
Weekend cortisol *	6 (26)
Pregnancy trimester	
First trimester	3 (10.0)
Second trimester	16 (53.3)
Third trimester	11 (36.7)
Mean gestational age (wk)	22.2 ± 7.1
PSS ^c^	
High PSS	5 (16.7)
Moderate PSS	20 (66.7)
Low PSS	5 (16.7)

Abbreviations: Body mass index (BMI), area under the curve with respect to the ground (AUCg), Perceived Stress Scale (PSS). ^a^ Household income was stratified and rounded to the nearest USD 1000 based on classification of wealth in the U.S. ^b^ Race and ethnicity were self-reported using separate categories for each according to the 1997 Office and Management and Budget guidelines [45]. ^c^ Perceived Stress Scale scores between 0 and 13 were classified as low, between 14 and 23 as moderate, and between 27 and 40 as high. * Time points recorded for saliva collection had 5 missing values. Weekday and weekend were averaged over the 2-day period.

**Table 2:** Substance and alcohol use among participants (N = 30).

Variables	Frequency (%)/Mean ± SD
Alcohol intake during pregnancy	
Less than or equal to 3 drinks per week	1 (13.3)
>3 drinks per week	1 (3.3)
Do not drink at all	28 (93.3)
Alcohol intake 3 months before pregnancy	
1 to 3 drinks a week	8 (26.7)
14 drinks or more a week	1 (3.3)
4 to 7 drinks a week	2 (6.7)
Less than 1 drink a week	10 (33.3)
No drink at all	9 (30.0)
Use of cigarettes 3 months before pregnancy per day	
1 to 5 cigarettes	2 (6.7)
6 to 10 cigarettes	2 (6.7)
Not all	26 (86.7)
Smoking cigarettes during pregnancy	
Do not smoke	30 (100)
Vaping of nicotine 3 months before pregnancy	
1 day a week or less	1 (3.3)
2–6 days a week	4 (13.3)
More than once a day	2 (6.7)
I didn’t vape nicotine then	23 (76.7)
Vaping of nicotine now (during pregnancy)	
I don’t vape	30 (100)
Vaping of marijuana/cannabis now	
I don’t vape	30 (100)

One drink = 12 oz beer, 5 oz wine, or 1.5 oz spirit; one pack = 20 cigarettes. Vape includes vape pens, e-hookahs, hookah pens, e-cigars, e-pipes, and battery-powered devices that use nicotine liquid rather than tobacco leaves and produce vapor instead of smoke.

**Table 3:** Association between vitamin D concentration and cortisol concentration among pregnant women (N = 30).

Cortisol Assessments	Vitamin D (25(OH)D)		
	β	(95% CI)	*p*-Value
Waking cortisol			
Unadjusted	−0.002	(−0.012, 0.008)	0.69
Adjusted ^a^	0.014	(−0.014, 0.016)	0.72
Cortisol 30 min after waking			
Unadjusted	−0.004	(−0.013, 0.006)	0.40
Adjusted ^a^	−0.003	(−0.015, 0.009)	0.96
Cortisol 45 min after waking			
Unadjusted	0.002	(−0.007, 0.010)	0.70
Adjusted ^a^	0.003	(−0.007, 0.014)	0.91
Cortisol 60 min after waking			
Unadjusted	0.002	(−0.007, 0.012)	0.62
Adjusted ^a^	0.004	(−0.008, 0.015)	0.91
Bedtime cortisol			
Unadjusted	−0.004	(−0.019, 0.011)	0.61
Adjusted ^a^	0.001	(−0.016, 0.020)	0.62
Cortisol AUC_g_			
Unadjusted	0.093	(−0.054, 0.241)	0.21
Adjusted ^a^	2.987	(−7.269, 13.244)	0.57
Cortisol awakening response			
Unadjusted	−0.008	(−0.012, 0.008)	0.53
Adjusted ^a^	0.002	(0.001, 0.006)	0.71

Abbreviations: β—Beta Coefficient; CI—Confidence Interval; AUC_g_—Area Under the Curve with response to the ground. ^a^ Adjusted model included covariates of age, pre-pregnancy BMI, race and ethnicity, and gestational age.

**Table 4:** Odds of vitamin D deficiency in relation to high cortisol concentration among pregnant women (N = 30).

Risk of Having Elevated Cortisol (Ref. = Optimum Vitamin D)	OR (95% CI)	*p*-Value
Ref: Optimum Vitamin D	1	
Waking Cortisol		
Unadjusted	0.79 (0.09, 5.61)	0.81
Adjusted *	0.07 (0.04, 0.09)	0.83
Cortisol +30 min		
Unadjusted	0.21 (0.01, 1.83)	0.21
Adjusted *	0.15 (0.001, 2.12)	0.23
Cortisol + 45 min		
Unadjusted	1.09 (0.15, 9.79)	0.92
Adjusted	0.001 (0.001, 19.81)	0.75
Cortisol + 60 min		
Unadjusted	1.25 (0.17, 10.96)	0.82
Adjusted *	0.11 (0.10, 15.22)	0.88
Bedtime Cortisol		
Unadjusted	0.25 (0.012, 2.04)	0.27
Adjusted *	0.047 (0.010, 6.963)	0.60
Cortisol AUC_g_		
Unadjusted	1.63 (0.23, 2.04)	0.63
Adjusted *	1.11 (0.09, 14.86)	0.93
Cortisol Awakening Response (CAR)		
Unadjusted	0.62 (0.06, 3.70)	0.41
Adjusted *	0.01 (0.003, 0.005)	0.78

Abbreviation: OR—Odds Ratio; CI—Confidence Interval. Adjusted model (*): Model was adjusted for covariates of maternal age, gestational age, pre-pregnancy BMI, and race and ethnicity.

**Table 5:** Pearson’s correlation matrix for perceived stress scores (PSSs), cortisol, Household Food Security Survey Module (HFSSM), and vitamin D.

	1	2	3	4
1. PSS	1.00	0.13	0.44	0.15
2. Cortisol *	0.13	1.00	0.40	0.23
3. HFSSM	0.44	0.399	1.00	0.28
4. Vitamin D	0.15	0.24	0.23	1.00

* The cortisol parameter used was the area under the curve with respect to the ground (AUCg).

## Data Availability

The data presented in this study are openly available in [PHANData verse] at [https://doi.org/10.7910/DVN/XKXPGH].

## Abbreviations

The following abbreviations are used in this manuscript:

AUC_g_	Area Under the Curve with Respect to the Ground
BMI	Body Mass Index
CAR	Cortisol Awakening Response
ELISA	Enzyme-Linked Immunosorbent Assay
PHAN	Prenatal Health and Nutrition Study
VDD	Vitamin D Deficiency
WIC	Women Infants and Children

## Appendix A

### Prenatal Supplementation and Vitamin D Status During Pregnancy

Figure A1 shows a relatively lower intake of prenatal supplements reported among participants in this study when compared to Figure A2. Overall, it is observed in both figures that with or without the reported intake of prenatal supplements, most of the participants remained classified as being vitamin D insufficient and deficient.
